# ‘Low-Plane’ Fractures of the Distal Humerus in Elderly Patients with Osteoporosis Show High Postoperative Complication Rates

**DOI:** 10.3390/jcm13216297

**Published:** 2024-10-22

**Authors:** Isabella Kuhn, Sophia S. Goller, Wolfgang Böcker, Boris M. Holzapfel, Daniel P. Berthold, Fabian Gilbert, Elisabeth Boehm

**Affiliations:** 1 Department of Orthopaedics and Trauma Surgery, Musculoskeletal University Center Munich (MUM), University Hospital, LMU Munich, 80336 Munich, Germany; 2 Department of Radiology, University Hospital, LMU Munich, 81377 Munich, Germany; 3 Schoen Clinic Munich Harlaching, 81547 Munich, Germany

**Keywords:** osteoporosis, distal humeral fracture, bone mineral density, computed tomography, dual-energy X-ray absorptiometry

## Abstract

**Background:** This study aimed to investigate the fracture patterns and complexity of distal humerus fractures with high-resolution computed tomography (CT) as a function of Dual-Energy X-ray absorptiometry (DXA)-derived bone marrow density (BMD) measurements in an elderly patient cohort. **Methods**: A retrospective chart review was conducted on patient data collected at a Level I trauma center between January 2007 and January 2022. Inclusion criteria comprised patients aged ≥40 years with a confirmed distal humerus fracture as demonstrated by CT. Additionally, patients were included if they underwent DXA. Patient demographics and detailed information regarding the surgical treatment and trauma mechanism were retrieved from the institutional databank. Fractures were classified as either ‘low-plane’ distal humeral fractures or ‘non-low-plane’ distal humerus fractures. Furthermore, the fracture patterns were classified according to established classification systems. Intra- and postoperative complication and revision rates were analyzed. **Results**: A total of 41 patients (30 women; mean age 74 ± 13 years) were enrolled. Low-energy trauma was sustained by 68% of the patients. The remaining 32% of the fractures involved medium-energy trauma. A total of 62% of the patients underwent primary osteosynthesis, while 30% of patients were initially treated with an external fixator. ORIF was performed in 89% of cases and, in the majority, double-plate osteosynthesis was used (76%). An olecranon osteotomy was performed in 30% of cases. A total of 5% of cases received total elbow arthroplasty, and 10% of cases were treated conservatively. A total of 61% of patients had osteoporosis, 24% of patients had osteopenia, and 15% of patients had a normal BMD with an overall mean T-score of −2.4. Most of the fractures were complex (including 61% Type C fractures). A total of 66% of cases were considered as ‘low-plane’ fractures. Postoperative complications occurred in 11% of patients (64% of cases among ‘low-plane’ fractures). Revision surgery was required in 20% of cases. **Conclusions**: The consecutive series of patients showed a high incidence of ‘low-plane’ fractures. However, no statistical significance was found between the BMD and fracture complexity. The very distal ‘low-plane’ fractures showed a high complication rate, which was aggravated by osteoporotic bone conditions. These findings highlight the need for future research with larger patient samples to better understand the relationship between the BMD, fracture complexity, and outcomes in patients with ‘low-plane’ fractures in order to reduce complications and improve clinical outcomes.

## 1. Introduction

Distal humerus fractures, occurring in 2–6% of the adult population, constitute a significant portion of humerus fractures, with distinct patterns observed in different age groups [1,2,3]. These fractures typically result from the following two distinct trauma mechanisms: high-energy trauma in young patients and low-energy falls, predominantly observed in older females [4,5,6].

Choosing the appropriate treatment involves considering factors including the complexity of the fracture, physical outcome requirements, age, and general health status. While open reduction and internal fixation (ORIF) remains the gold standard for younger patients, the elderly population presents unique challenges. Anatomic repair, often unsatisfactory or associated with high complication rates in the elderly [7,8], encounters difficulties in cases of osteoporotic bone and may lead to joint instability, functional impairment, or the aseptic loosening of osteosynthetic material [9,10]. Unfortunately, up to 30% of older patients may find the ORIF approach unsatisfactory due to persistent pain, implant failure, or the need for revision [7,8].

As the current demographic shift contributes to an apparent increase in distal humerus fractures, particularly in the presence of osteoporosis, there is a need for a deeper understanding of fracture patterns and their implications on therapeutic outcomes. To date, limited information exists regarding the correlation between distal humerus fractures and osteoporosis. As these fracture types are very rare, clinical studies are limited, especially in geriatric patient cohorts, resulting in a lack of conformity among existing research [8,11,12].

Given this background, the aim of this retrospective study was to investigate fracture patterns, particularly in the distal plane, and to assess the complexity of distal humerus fractures as a function of Dual-Energy X-ray absorptiometry (DXA)-derived bone marrow density (BMD) measurements in patients aged ≥40 years from a Level I trauma center. The hypotheses of this study were that (1) osteoporosis is associated with more complex ‘low-plane’ fractures and (2) these complex fractures exhibit higher complication rates than the more proximally localized distal humerus fractures.

## 2. Methods

### 2.1. Patient Selection

This retrospective study was approved by the local Ethics Committee of the Medical Faculty, University of Munich (project number 22-0799, 10 October 2022), and was conducted in accordance with national ethical guidelines and the Helsinki Declaration of 1964 and its later amendments.

**Figure 1:**
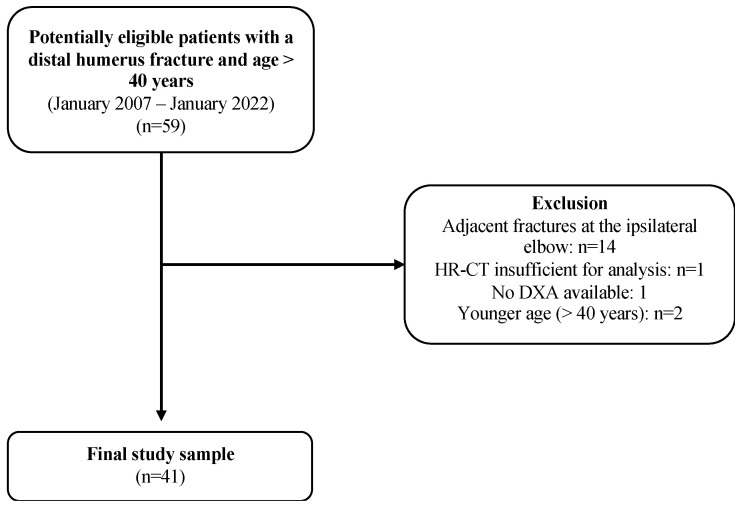
Flowchart illustrating the patient selection process. From n = 59 potentially eligible patients, 18 patients had to be excluded during the selection process, resulting in a final study sample of 41 patients. HR-CT, high-resolution computed tomography; DXA, dual-energy X-ray-absorptiometry.

A retrospective chart review was conducted on patient data collected at a Level I trauma center between January 2007 and January 2022 (Figure 1). Inclusion criteria comprised patients aged ≥40 years with a confirmed distal humerus fracture as demonstrated by high-resolution computed tomography (CT). Additionally, patients were included if they underwent DXA of the lumbar spine and the proximal femur at the same institution, with a maximum time interval of three months from the fracture diagnosis. Patients with adjacent fractures of the ipsilateral humerus were excluded. Patient-specific information was retrieved from the institutional databank, including the patient’s medical history.

Fractures were treated either surgically or conservatively. Surgery included osteosynthesis with either single- or double-plate techniques, with additional prior fixation, if necessary, or total arthroplasty.

The association between imaging characteristics and postoperative complication rates was assessed via subgroup analyses. According to imaging assessment, patients were either assigned to the ‘low-plane’ or ‘non-low-plane’ group. The exact surgical technique, the duration of the hospital stay, the treatment details, and the presence of complications and revision surgeries were meticulously documented for each group.

The trauma mechanisms leading to fractures were classified into the following three categories: low-, medium-, and high-energy trauma. Low-energy trauma, often seen in the elderly, was described as a fall from a standing position, such as a tripping fall. Medium-energy trauma included incidents like bicycle falls, falls on ice or stairs, and car accidents at walking speed. High-energy trauma included higher-speed car crashes or falls from a height greater than 2 m, resulting in polytraumatic injuries. Open fractures were classified according to the Gustilo and Anderson Classification [13].

### 2.2. Bone Mineral Density Assessment

Spine and femur BMD measures were acquired on two DXA scanners (Lunar Prodigy and Lunar Prodigy Advance, GE Healthcare, Chicago, IL, USA) according to current standards using T-scores [14]. A T-score of less than −1 was defined as ‘normal bone density’, while ‘osteopenia’ was characterized by a T-score of less than -1 and greater than −2.5. ‘Osteoporosis’ was designated for a T-score of less than −2.5. Patients’ 25-Hydroxyvitamin-D (25(OH)D levels were recorded. A vitamin D level above 30 ng/mL was considered physiological [15].

### 2.3. Conventional CT

CT data were acquired in our institution using the following scanners: the GE Revolution and GE Optima, GE Healthcare; the Somatom Definition Edge, Somatom Drive, and Somatom Force, and Siemens Healthineers. Multiplanar reconstructions were conducted with a standard bone kernel (170H/YB, 3 mm slices). Clinical scan parameters were set as follows: collimation, 0.6 mm; pixel spacing, 0.56 mm; pitch factor, 0.8; peak tube voltage, 120 kV; modulated tube current, 102–132 mA.

### 2.4. CT Image Analysis

Fractures were systematically categorized according to the established Arbeitsgemeinschaft für Osteosynthesefragen (AO) classification [16] and Dubberley’s classification [17] on CT scans. A single surgeon (*E.B.*) evaluated all of the CT images and performed measurements within the local Picture Archiving and Communication System (PACS).

**Figure 2:**
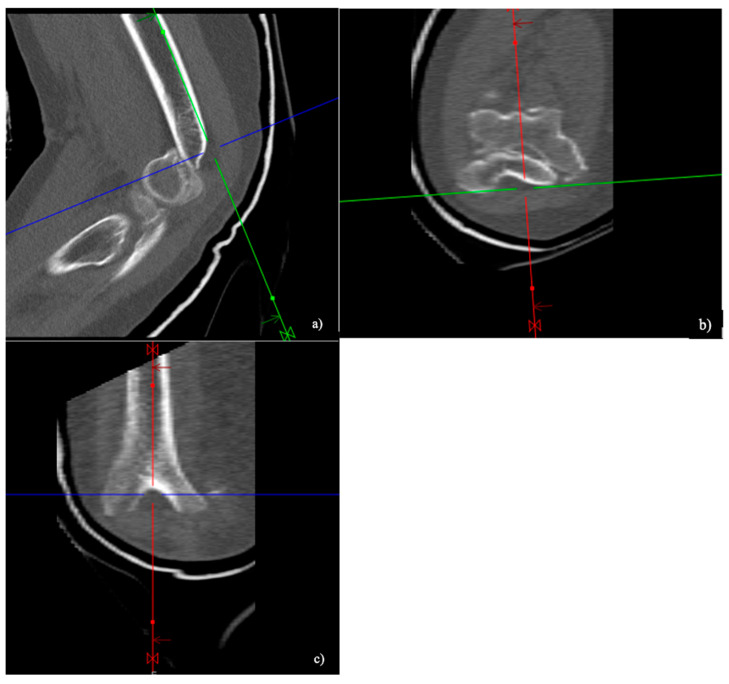
Computer tomographic (CT) images of a ‘low-plane’ distal humerus fracture in the sagittal (**a**), axial (**b**), and coronal (**c**) reconstruction.

**Figure 3:**
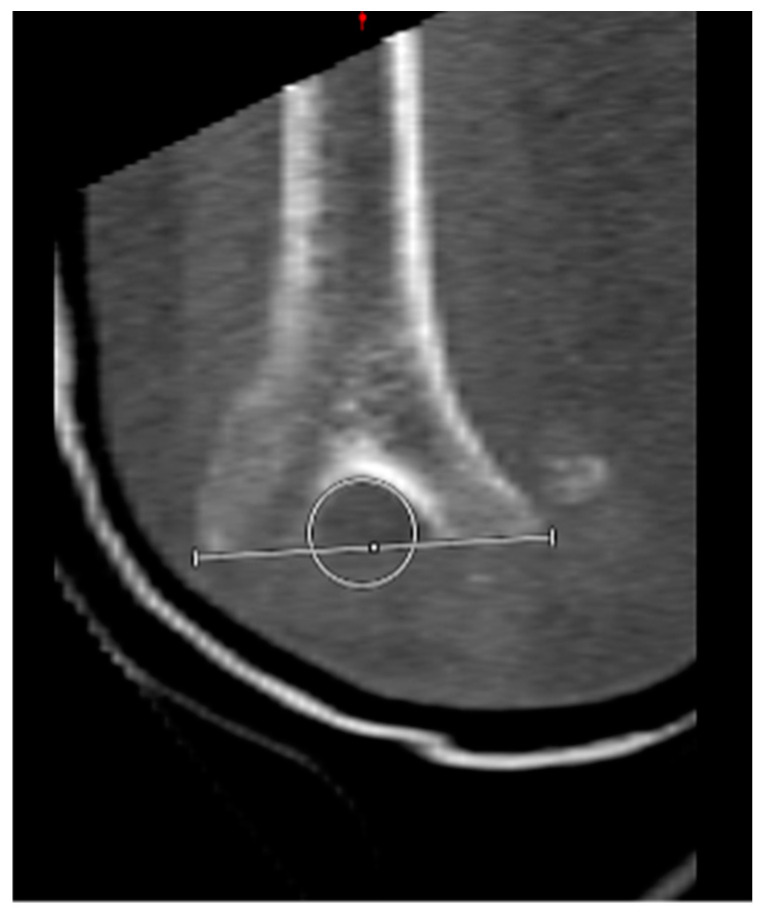
Positioning of a “circle of best fit” over the olecranon fossa. Definition of a ‘low-plane’ fracture for fractures located in the distal half of the circle.

**Figure 4:**
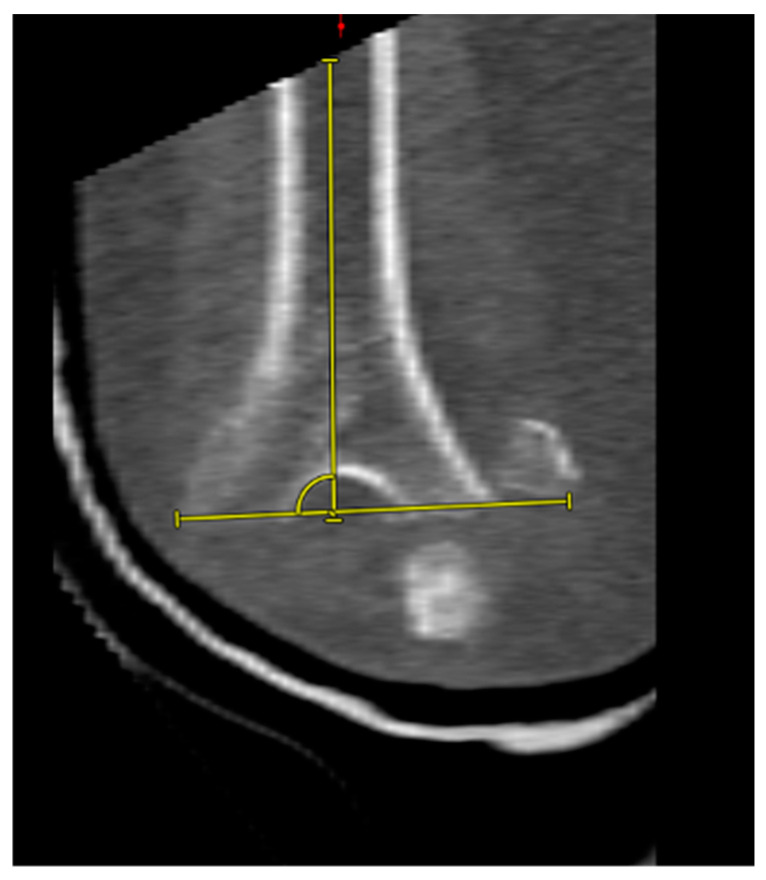
Fracture angle measurement towards the radial side.

Based on their morphology, the fractures were classified as either ‘low-plane’ distal humeral fractures or ‘non-low-plane’ distal humerus fractures (Figure 2). To the best of our knowledge, the term ‘low-plane’ fractures is used in the literature; a coherent definition is, however, currently lacking. In this study, the fractures were defined as ‘low-plane’ if they were located in the distal half of a ‘circle of best fit’, positioned over the olecranon fossa in the coronal plane (Figure 3). Analogous to this, the fractures located more proximally than the ‘low-plane’ fractures were classified as ‘non-low-plane’ fractures. The fracture angle in relation to the humeral shaft axis, aligned radially, was measured (Figure 4), as well as the number of fragments and columns affected.

### 2.5. Statistical Analysis

Descriptive statistics, encompassing the mean and standard deviation for continuous variables, and the frequency and proportion for categorical variables, were computed. Statistical analyses were conducted using SPSS Statistics (v28, IBM Corporation, Armonk, NY, USA). The observed results were considered statistically significant at an alpha level of ≤0.05.

## 3. Results

### 3.1. Patient Characteristics

During the inclusion period, 41 patients (30 females, mean age 74 ± 13 years, range 41–99 years) presented with a distal humerus fracture and fulfilled the inclusion criteria. Detailed demographic data of the patients are displayed in Table 1.

### 3.2. Surgical Treatment

Regarding treatment, 37 (90%) of the fractures were managed surgically, with direct osteosynthesis performed in 23 cases (62%). In 11 cases (30%), patients initially underwent external fixation before receiving subsequent operative treatment. ORIF was performed in a total of 33 cases (89%), with 25 cases (76%) fixed by double plating and 4 patients (12%) by single-plate osteosynthesis and by screw osteosynthesis. For the double-plate osteosynthesis, the plates were arranged orthogonally in 15 cases (60%) and parallel in 10 cases (40%). An olecranon osteotomy was performed in 11 cases (30%).

In two cases (5%), only a fragment resection was performed, and in another two cases (5%), the fracture was treated with endoprosthetic joint replacement. A conservative approach was chosen for four patients (10%) due to age, comorbidities, or a low functional demand.

In the ‘low-plane’ group, 4 patients (14%) were treated conservatively, 2 patients (7%) underwent fragment resection, 16 patients (57%) had a direct osteosynthesis, and 5 patients (18%) underwent first external fixation and then open reduction and internal fixation. One patient (4%) with a ‘low-plane’ fracture received an elbow prosthesis.

All of the patients with a more proximally located fracture were treated surgically, where 7 patients (54%) underwent an osteosynthetic procedure, 5 patients (38%) received a fixator prior to osteosynthesis, and 1 patient (8%) received an elbow prosthesis.

The average clinical stay in the ‘low-plane’ group was 10 ± 6.3 days versus 9 ± 6.3 days in the ‘non-low-plane’ group.

### 3.3. Bone Mineral Density

**Figure 5:**
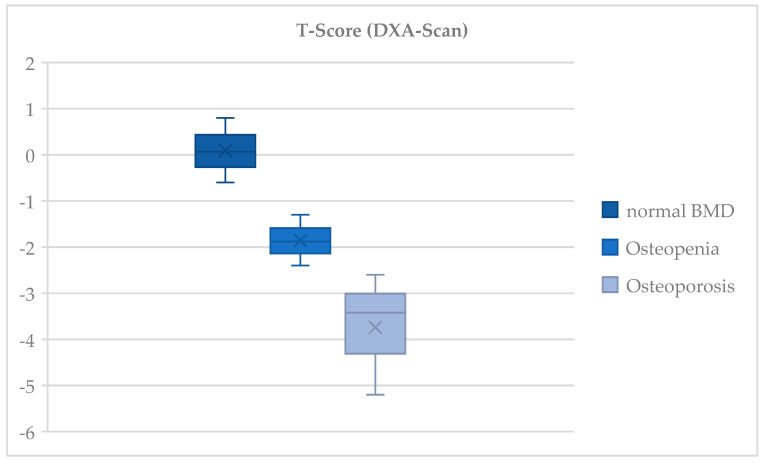
Distribution of T-scores for the bone mineral density according to the Dual-Energy X-ray absorptiometry. Presentation of T-scores according to the Dual-Energy X-ray absorptiometry (DXA)-derived bone mineral density (BMD) measurements. A T-score of less than −1 was defined as ‘normal bone density’, while ‘osteopenia’ was characterized by a T-score of less than −1 and greater than −2.5. ‘Osteoporosis’ was defined for a T-score of less than −2.5.

According to the DXA scans, 35 patients (85%) exhibited a reduced BMD. Specifically, 25 patients (61%) had osteoporosis and 10 patients (24%) had osteopenia. Overall, the mean T-score was −2.5 ± 1.4 (range −5.2 to 0.8). The T-score distribution in regard to the BMD is shown in Figure 5. The mean vitamin D level was 22.5 ± 13.7 (range 10.0–62.2) ng/mL.

Among those patients presenting with a ‘low-plane’ fracture, 19 patients (68%) had reduced bone quality, with osteoporosis in 20 cases (71%) and osteopenia in 8 of these cases (19.0%). Of those patients with a ‘non-low-plane’ fracture, five persons (38%) had osteoporosis, five (38%) had osteopenia, and three (23%) had a normal BMD. The average T-score in the ‘low-plane’ group was −2.7 ± 1.4 and it was −2.1 ± 1.5 in the ‘non-low-plane’ group, respectively.

### 3.4. Classification of Fractures

In 24 cases (59%), the fractures consisted of four or more fragments. Both the medial and lateral columns were affected in 37 (90%) cases, while only the lateral column was involved in 3 cases (7%), and only the medial column was involved in 1 case (3%). The average fracture angle measured was 93° ± 17° (range of 53–128°). ‘Low-plane’ fractures were observed in 28 patients (68%), while ‘non-low-plane’ fractures were seen in 13 patients (32%). Detailed information is shown in Table 1.

**Figure 6:**
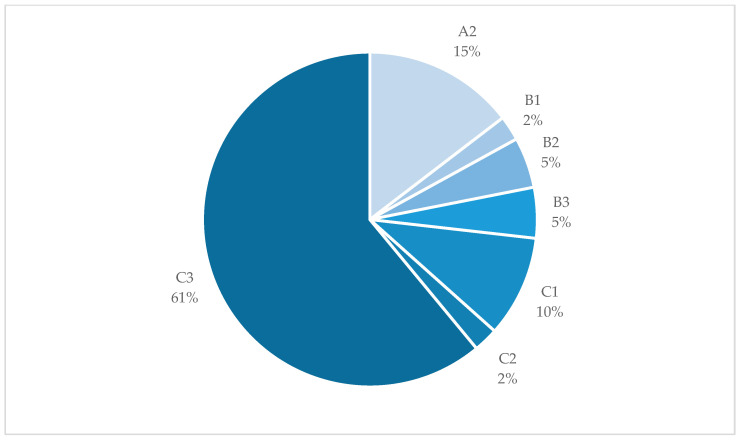
Fracture types according to the AO Classification. Pie chart showing the fracture types classified according to the AO Classification of distal humerus fractures in the study group. The majority of fractures were Type C3 fractures.

Assessment of the fractures according to the AO Classification revealed a predominance of Type C3 fractures (61%; n = 25) (Figure 6).

Among all of the fractures, six (15%) were identified as open, presenting as grade 1 (n = 4) or grade 2 (n = 2) according to the Gustilo and Anderson Classification. The remaining 35 fractures (75%) were considered closed. Looking at the ‘low-plane’ and ‘non-low-plane’ fractures in detail, a higher incidence of more complex C3 fractures was found in the distal, ‘low-plane’ area, with 14 (34%) versus 10 (24%) in the more proximal region, respectively. Another five (18%) of the ‘low-plane’ fractures were classified as simpler A2 fractures, versus only one case (2%) in the ‘non-low-plane’ fracture group. Except for the A1 and C2 fractures, ‘low-plane’ fractures were uniformly present compared to the more proximal fractures.

### 3.5. Complications

The overall complication rate amounted to 27% (n = 11). Of those cases, nine (82%) were observed in patients with complex C3 fractures, along with B2 (9%) and B3 (9.0%) (Dubberly 3B) fractures. Among the postoperative complications, seven (64%) occurred in the presence of a ‘low-plane’ fracture. A complication rate of 36% (n = 4) was found in the ‘non-low-plane’ fracture group. Osteosynthesis-related complications such as screw dislocation and the loosening or breakage of the plate occurred in three cases (10.0%) in the ‘low-plane’ group compared to one patient in the ‘non-low-plane’ group (3%) (*p* = 1.0). The majority of patients who encountered complications demonstrated reduced bone quality, specifically, osteoporosis in five cases (45%) and osteopenia in four cases (36%). Two patients showed physiological bone quality according to the DXA measurements. Complications occurred in four cases (36%) after intraoperative olecranon osteotomy. One patient suffered from a re-fracture after osteotomy of the olecranon. The total revision rate was 20% (n = 8).

Observed complications are summarized in Table 2.

## 4. Discussion

The most important finding of this study was the high incidence of more distal, ‘low-plane’ fractures in the presence of osteoporosis in patients aged ≥40 years. In clinical practice, elderly patients often present with a multifragmented fracture pattern located in the distal part over the olecranon fossa, which was classified as a so-called ‘low-plane fracture’.

Distal humerus fractures in geriatric patients present unique challenges, often complicated by factors such as osteoporosis and frailty. Currently, surgical interventions, particularly ORIF, are considered the gold standard. However, the aging population poses increased risks of complications. Compromised bone structure, vulnerable soft tissues, comorbidities, reduced compliance, and mobility contribute to higher complication rates, reaching up to a complication rate of 35% in the literature [8,11,18].

In this study group, a notably high rate of complications emerged in the ‘low-plane’ fractures. When comparing the findings of this study to the current literature, Charissoux et al. in 2013 and Varecka et al. in 2017 demonstrated that the complexity of fractures, particularly in elderly women, often involves a multifragmented pattern located in the distal part over the olecranon fossa [19,20]. The data from this study align with the current literature, where Charissoux et al. demonstrated a higher incidence of Type C3 fractures in a population over 65 years old, emphasizing a trend toward distal humerus fractures resulting from low-energy trauma, especially in elderly women.

Osteoporosis, an underdiagnosed pathology in an aging society, contributes to the complexity of therapy [21]. Sustained osteoporotic fractures increase the risk of subsequent fractures, reducing the independence of the patients and causing a significant socioeconomic burden [22]. Even with surgical treatment, complication rates are generally higher than with normal bone conditions. In this studied group, 81% of patients with reduced bone density diagnosed with DXA experienced complications. Notably, a 36% complication rate occurred after intraoperative osteotomy of the olecranon. Moreover, the rarity of distal humerus fractures conceals an increasing trend in the elderly, particularly impacting females. Osteoporosis exacerbates outcomes, challenging the conventional gold standard of surgical anatomic repair.

Alternatively, total elbow arthroplasty (TEA) may be seen as an alternative in this highly challenging patient cohort. However, the ongoing debate between osteosynthesis and arthroplasty for geriatric patients is evident in the literature [23,24]. While McKee et al. suggest joint replacement as a viable alternative, Moursy et al. advocate for surgical osteosynthesis despite the high complication rate, which is often clinically asymptomatic [11,12]. Considering the higher complication rate in the ‘low-plane’ group and in osteoporotic conditions, anatomical reconstruction should be attempted first to avoid a possible fatal outcome after first-line arthroplasty. If arthroplasty is considered an option, olecranon osteotomy should be avoided [25].

In patients with a low functional demand or in cases of advanced frailty, adopting the “bag of bones” approach as a conservative treatment method may be considered [26]. This strategy aims to avoid the substantial surgical risks associated with ORIF or TEA.

The ongoing discourse on the most effective approach—ORIF, TEA, or conservative treatment—underscores the necessity for continued research and re-evaluation in this unique patient population.

The limitations of this study include a limited patient cohort of this rare pathology and its retrospective analysis. Moreover, only patients who simultaneously underwent DXA and CT scans were included, leading to a potential selection bias. Even though the institutional policy strongly enforces osteoporotic surveillance and DXA scans in selected patient groups, some potential patients may have been missed, as they did not undergo DXA and no information on their BMD values was available. Additionally, the data were collected at a single center only. Due to the narrow inclusion criteria, there was, however, no group with available DXA scans for comparison in which the normal BMD could be assumed. Therefore, only descriptive statistics could be used. Furthermore, the studied patients were patients aged ≥40 years who might have had other comorbidities affecting the treatment choice and outcome. Another limitation of this study is, however, that imaging data were reviewed by only one surgeon, and patients were not specifically clinically assessed for this study, so no correlation could be made between ‘low-plane’ fractures and long-term clinical outcomes or patient satisfaction. Nevertheless, the data from this study show a trend towards more ‘low-plane’ fractures in the presence of osteoporosis, which should be the subject of further research, especially in light of the ongoing demographic changes.

## 5. Conclusions

This consecutive series of patients showed a high incidence of ‘low-plane’ fractures. However, no statistical significance was found between the BMD and fracture complexity. Yet, in this cohort, the distal ‘low-plane’ fractures showed a high complication rate, aggravated by osteoporotic bone conditions. These findings highlight the need for future research with larger patient samples to better understand the relationship between the BMD, fracture complexity, and outcomes in patients with ‘low-plane’ fractures in order to reduce complications and improve clinical outcomes.

## Figures and Tables

**Table 1:** Demographics of ‘low-plane’ fractures versus more proximally located fractures.

	‘Low-Plane’	‘Non-Low-Plane’
Number of patients	28	13
Age (years)	75 ± 13 * (41–99) **	72 ± 12 * (50–92) **
Male	7	4
Female	21	9
Low-energy trauma	20 (71%)	8 (62%)
Medium-energy trauma	8 (29%)	5 (39%)
**AO-Classification**		
A1	0	0
A2	5 (18%)	1 (2%)
B1	1 (2%)	0
B2	2 (5%)	0
B3	1 (2%)	1 (2%)
C1	4 (10%)	0
C2	0	1 (2%)
C3	14 (34%)	10 (24%)

This table provides descriptive data on patient demographics and fracture characteristics. * Data are given as mean ± standard deviation. ** Data are given as a range.

**Table 2:** Postoperative complications.

Complications	‘Low-Plane’	“Non-Low-Plane’
Osteosynthesis failure	3 (10%)	1 (3%)
Ulnar nerve injury	1 (3%)	-
Instability	-	1 (3%)
Soft tissue irritation	2 (5%)	1 (3%)
Arthrofibrosis	-	1 (3%)
Heterotopic ossification	1 (3%)	-
Failure after olecranon osteotomy	2 (18%)	2 (18%)

This table provides descriptive data summarizing the postoperative complications. Categorical variables are presented as counts with percentages in parentheses.

## Data Availability

The authors confirm that the data supporting the findings of this study are available within the article.
